# Evaluation and comparison of aluminum-coated pumice and zeolite in arsenic removal from water resources

**DOI:** 10.1186/1735-2746-9-38

**Published:** 2012-12-31

**Authors:** Simin Nasseri, Masoumeh Heidari

**Affiliations:** 1 Department of Environmental Health Engineering, School of Public Health and Center for Water Quality Research, Institute of Environmental Research, Tehran University of Medical Sciences, Tehran, Iran; 2 Department of Environmental Sciences, Sanandaj Branch, Islamic Azad University, Sanandaj, Iran

**Keywords:** Arsenic adsorption, Water resources, Aluminum-coated pumice, Aluminum-coated zeolite

## Abstract

In this research the potential of aluminum-coated pumice and zeolite in arsenic, As (V) removal was investigated and compared. Scanning Electron Microscopy (SEM), X-Ray Diffraction (XRD) and X-Ray Flaorescence Spectrometry (XRF) were carried out to determine the properties of the adsorbents. Several parameters including adsorbent dosage] pH, contact time, and initial As(V) concentration were studied. The optimum pH obtained for both adsorbents was pH = 7. As(V) adsorption by both adsorbents followed the Freundlich isotherm (for aluminum-coated pumice and zeolite respectively with R^2^ > 0.98 and R^2^ > 0.99). The obtained data from kinetics showed that the pseudo-second order model could better explain As(V) adsorption for both aluminum-coated pumice and zeolite (R^2^ > 0.98 and R^2^ > 0.99 respectively). Because of low cost, both adsorbents may be economically used, but aluminum-coated zeolite showed high efficiency of, due to its porosity and surface area. More than 96% of As(V) with initial concentration of 250 μg/L was removed by 10 g/L aluminum-coated zeolite at pH = 7 and in 60 minutes to achieve As(V) concentration of 10 μg/L, while only 71% of As(V) could be removed by aluminum-coated pumice.

## Introduction

The presence of arsenic in water resources has become an important problem to human safety because of its toxicity [1,2]. Long–term exposure to inorganic arsenic in drinking water leads to adverse health effects such as pigmentation, depigmentation, keratosis and cancer of the bladder, lungs, skin, kidney, nasal passages, liver and prostate [3]. An arsenic concentration of 10 μg/L has been recommended by World Health Organization as a guideline value for drinking water [4].

Arsenic exists in natural waters in both inorganic and organic forms. The inorganic form of arsenic is more toxic than its organic form. Inorganic arsenic exists in natural water resources in two oxidation states: arsenite, As(III) and arsenate, As(V) [5,6]. Arsenite is dominant in more reduced conditions, where as arsenate is dominant in oxidizing condition. As(III) is approximately ten times more toxic and mobile than As(V) [7,8], but As(III) can be converted to As(V) by several oxidants such as chlorine compounds, ozone, permanganate or manganese oxide and Fenton’s reagent [1,9]. As(V) may be adsorbed more strongly than As (III) on to the solid phase.

Arsenic can be removed from water resources by several methods such as precipitation, anion exchange, reverse osmosis, coagulation, ultrafiltration and adsorption [10]. Adsorption process seems to be the most promising method based on its removal efficiency, low cost, availability and easy operation and the potential for removing trace amount of toxic elements from large volumes of solutions [11,12]. Adsorption by activated alumina, activated carbon, rare earth oxides, manganese green sand and natural stones such as zeolite have been used to remove arsenic from water [2,13,14].

Impregnation and coating with chemicals enhances the sorption capacity of natural adsorbents [15]. Tripathy and Raichur [15] studied the adsorption of As(V) by activated alumina and showed that the efficiency of activated alumina increases by impregnating with alum.

Pumice and zeolite particles are previously assessed in arsenate adsorption. Pumice is a porous igneous rock; it is formed during explosive volcanic eruptions, when liquid lava is emitted into the air as a froth containing mass of gas bubbles [16]. Zeolites are hydrated alkali or alkaline earth metals aluminum silicates with a crystal structure and can have a big empty space inside. Zeolites have very large surface areas and their ion exchange capacity are high [17].

In this study, As(V) removal from water resources using aluminum-coated pumice (ACP) and aluminum-coated zeolite (ACZ) as new adsorbents were investigated and compared. Also the effects of parameters including adsorbent doses, pH, contact time, initial As(V) concentration and interfering ions are studied.

## Materials and methods

### Preparation of solutions

Stock solutions of As (V) were prepared by dissolving Na_2_HAsO_4_.7H_2_O in double distilled water. Aluminum solution for coating natural particles of pumice and zeolite stones were prepared by dissolving Al_2_ (SO_4_)_3_18H_2_O. All glassware and bottles were washed by 1 N HNO_3_ and rinsed with double distilled water before usage. All chemicals were purchased from Sigma Aldrich (Spain).

### Preparation of aluminum-coated pumice and zeolite [(ACP)], (ACZ)

Prior to the coating of aluminium on the surface of the pumice and zeolite stones, these stones were crushed by a jaw crusher and were screened by a sieve (Mesh No. 50). Sieved particles of the pumice were kept in 37% HCl for 24 h and were washed several times with double distilled water. Then, the particles of the pumice and zeolite stones were immersed in double distilled water for 24 h and were dried at 105°C in the oven for 14 h. In order to coat the particles of pumice and zeolite with alum, a solution of 0.5 M Al_2_ (SO_4_).16H_2_O was prepared. Afterwards, 50 g of pumice and zeolite particles with 150 mL of 0.5 M alum solution were added into two beakers separately and pH was adjusted to 11 by adding 10 M NaOH solution drop by drop, while stirring for 2 min. Thereafter, the beakers were placed in a static and stable state in laboratory temperature (25 ± 1°C) for 72 h and were dried at 110°C in the oven for 14 h. In order to remove traces of uncoated alum from the particles, the dried particles were washed again with double distilled water and were dried in the oven at 105°C for 14 h [18]. Then the chemical compositions of the pumice and zeolite were determined by X-Ray Fluorescence (XRF) spectrometry (Model: Thermo, ARL, ADVAN’X Series). Besides, the surface area of the adsorbents were revealed using Quntasorb surface area measurement apparatus.

### Batch experiments

All experiments were conducted in batch mode and in a series of 250 mL conical flasks. Parameters were studied in the range of pH (3–11), adsorbent dosage of (2.5-60 g/L), initial As(V) concentration of (50, 250 and 2000 μg/L) and contact time (0–200 min). The conical flasks containing As(V) solution and the various doses of adsorbents were separately mixed by orbital shaker at 200 rpm in constant temperature (25 ± 1°C). At the end of the adsorption process, the samples were filtered through 0.45 μm membrane filter, centrifuged at 3000 rpm and analyzed by atomic adsorption spectrophotometer (Model: 220 Varian, Australia). Afterwards, residual As (V) concentration was calculated by the equation (1):

(1) $$q_{e}=\;C_{0}-\;C_{e}\times \frac{V}{M}$$

Where q_e_ is the amount of the adsorbate (mg/g), C_0_ is initial As (V) concentration (mg/L), C_e_ is the residual equilibrium As (V) concentration (mg/L), V is the volume of the solution (L) and M is adsorbent dosage (g).

The pH was adjusted by pH meter (Model: Suntex sp-701, Taiwan) with diluted 0.1 M HCl and 0.1 M NaOH solution. All experiments were duplicated and the means were reported.

### Kinetic experiments

Batch experiments were carried out to determine the time profiles of arsenic adsorption to ACP and ACZ. The samples were collected from the conical flask after 0, 1, 5, 10, 15, 20, 30, 40, 55, 70, 100, 130, 160 and 200 minutes, filtered, centrifuged and analyzed for arsenic concentrations.

The pseudo-first order and pseudo-second order models are the most popular kinetic models to study the adsorption equilibrium:The pseudo-first order model is described as equation 2 [19]:

(2) $$\frac{{\mathrm{dq}}_{t}}{\mathrm{dt}}=K_{1}\left(q_{e}-q_{t}\right)$$

Where, q_e_ and q_t_ are the amounts of adsorbent (mg/g) at equilibrium and time (min), respectively. K_1_ is the constancy of the adsorption rate (1/min).

Integration of equation (2) at the boundary of q_t_ = 0 at t = 0 and q_t_ = q_t_ at t = t leads to equation (3):

(3) $$\;\log\left(1-\frac{q_{t}}{q_{e}}\right)=-\frac{K_{1}}{2.302\;t}$$

The pseudo-second order model is written as equation 4 [20]:

(4) $$\frac{{\mathrm{dq}}_{t}}{\mathrm{dt}}=K_{2}{\left(q_{e}-q_{t}\right)}^{2}$$

Where K_2_ is the constancy rate (mg/g).The linear form of equation (4) at the boundary of qt = 0 at t = 0 and q_t_ = q_t_ at t = t can be described as equation (5):

(5) $$\frac{\text{t}}{q_{t}}=\frac{1}{K_{2}q_{e}^{2}}+\frac{1}{q_{e}t}$$

### Adsorption isotherm

The sorption isotherm experiments were carried out for several adsorbent doses ranging from 1.2–40 g/L at pH = 7 and a constant initial As (V) concentration of 250 μg/L. Thereafter, equilibrium times were deducted from the kinetic experiments and fixed in 24 h and the reaction mixtures were filtered, centrifuged and analyzed for arsenic concentrations. Finally, the equilibrium data were analyzed in accordance with Freundlich and Langmuir sorption isotherm models.

The non–linear equation of Freundlich isotherm model is as equation 6:

(6) $$q_{e}=K_{f}C_{e}^{\frac{1}{\text{n}}}$$

Where, K_f_ and n are Freundlich isotherm constants, which respectively show the adsorption capacity and intensity of adsorbent. The linear form of Freundlich equation is shown in equation (7):

(7) $$\log q_{e}=\log K_{f}+\frac{1}{\text{n}}\log C_{e}$$

Values of K_f_ and n are obtained from the slope and interception of a plot resulted from log q_e_ versus log C_e_.

The nonlinear form of Langmuir isotherm model is:

(8) $$q_{e}=\frac{q_{m}{\mathrm{bC}}_{e}}{1+{\mathrm{bC}}_{e}}$$

Where, q_m_ is the maximum capacity of the adsorbent and b is the Langmuir constancy. The linear form of Langmuir equation is as Equation 9:

(9) $$\frac{C_{e}}{q_{e}}=\frac{C_{e}}{q_{m}}+\frac{1}{{\mathrm{bq}}_{e}}$$

Values of q_m_ and b can be obtained from the slope and interception of a plot of C_e_ versus C_e_/q_e_.

## Results

### Characterizations of the adsorbent

**Figure 1:**
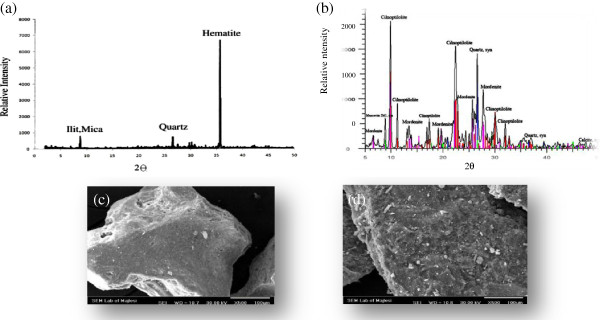
(a) X-ray diffraction spectrum of ACP, (b) X-ray diffraction spectrum of ACZ,(c) SEM image of ACP, (d) SEM image of ACZ.

The solid structures and photomicrography of the exterior surfaces of ACP and ACZ analyzed by using SEM are show in Figure 1. The results of the solid structure of the adsorbents which have been analyzed by XRD are also presented in Figure 1. Results of chemical composition of pumice and zeolite are given in Table 1.

**Table 1:** Chemical and physical composition of zeolite and pumice

**Zeolite (%)**	**Pumice (%)**	**Constituent**
63.12	51.45	SiO_2_
12.60	17.08	AL_2_O_3_
4.03	6.44	CaO
2.63	3.26	K_2_O
1.68	5.67	Na_2_O
1.67	6.32	Fe_2_O_3_
1.12	6.17	MgO
0.23	1.54	TiO_2_
0.19	0.66	P_2_O_5_
0.128	0.22	SrO
0.088	-	BaO
0.072	0.09	MnO
0.048	0.52	SO_3_
0.014	-	ZrO_2_
0.008	-	CuO
0.008	-	Rb_2_O
0.006	-	ZnO
12.28	0.56	LOI^*^
1.00	0.91	Density ( g/cm^3^)
15	7	(m^2^/g) BET

* Loss on Ignition.

### The effect of the adsorbent dosage and initial As (V) concentration

**Figure 2:**
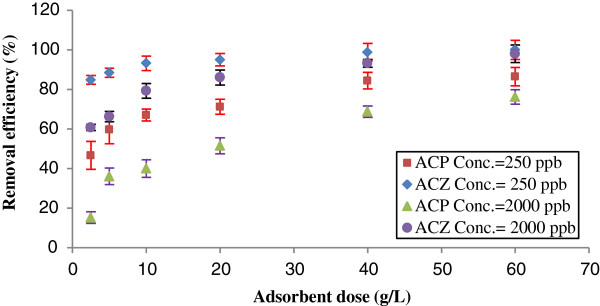
**The effect of the adsorbent doses and initial As(V) concentrations on As(V) removal, temperature: 24 ± 1°C and pH: 7.** Error bars correspond to confidence intervals of 95%.

The effect of different doses of adsorbents and initial As (V) concentration on removal percentage of As (V) by ACP and ACZ are shown in Figure 2.

### The effect of the solution pH

**Figure 3:**
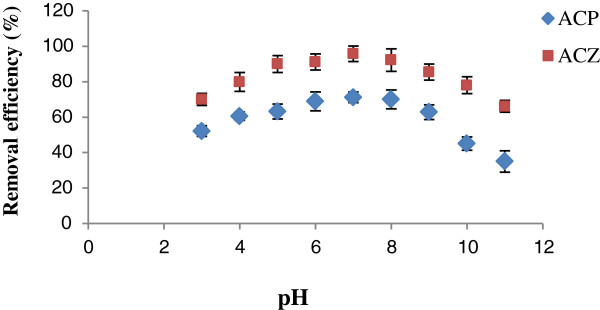
**The effect of pH variation on As(V)removal by ACP and ACZ: temperature = 24 ± 1°C, initial As (V) concentration = 250 μg/L and adsorbent dosage = 10 g/L.** Error bars correspond to confidence intervals of 95%.

The results of pH effect on As (V) adsorption onto ACP and ACZ with initial As (V) concentration of 250 μg/L are separately shown in Figure 3.

### The effect of contact time

The effect of contact time on As (V) adsorption by ACP and ACZ with initial As (V) concentration of 250 μg/L is shown in Table 2.

**Table 2:** The effect of contact time on As (V) adsorption by ACP and ACZ

**Time(min)**	**Removal (%) for ACP**	**Removal (%) for ACZ**
5	40.1	88.4
10	51.2	90
15	60.8	90.8
20	64.8	92.4
30	66.8	94.8
40	69.8	95.6
55	71.2	96
70	80	97.8
100	91.6	98.1
160	95.2	98.8

### Kinetics of the adsorption

**Figure 4:**
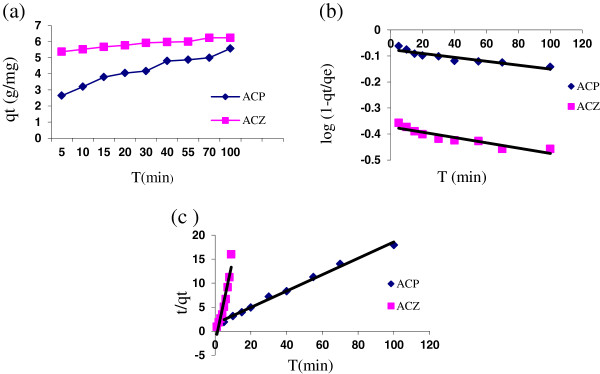
Fitting of the obtained data onto (a) pseudo-first order model, (b) pseudo-second order model: temperature = 24 ± 1°C, pH = 7; initial As (V) concentration = 250 μg/L and adsorbent doses = 10 g/L.

Batch experiments were carried out to determine the time profiles of arsenic adsorption on ACP and ACZ. In order to estimate the rate of adsorption, the adsorption kinetics of As(V) onto ACP and ACZ for three models were studied at different intervals of time and are shown in Figure 4. The parameters relating to the two kinetic models for both adsorbents are presented in Table 3.

**Table 3:** Parameters related to the kinetic models for ACP and ACZ adsorbents

**Models**	**K**_**1**_	**K**_**2**_	**K**_**m**_	**K**_**p**_	**q**_**e**_	**R**^**2**^
**Pseudo**–**first order**	*0.0010	–	–	–	*20	*0.80
	●0.0009				●09.58	●0.85
**Pseudo**–**second order**	–	*0.0128	–	–	*0.57	*0.98
		●0.0136			●6.26	●0.99

*ACP, ●ACZ.

### The adsorption isotherms

**Figure 5:**
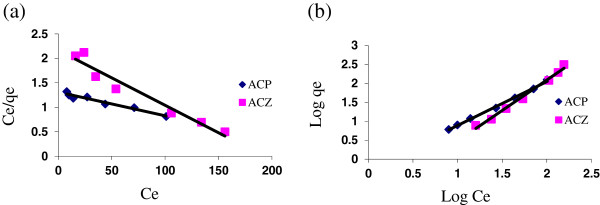
(a) Langmuir; (b) Freundlich isotherms plots for As (V) adsorption on ACP and ACZ ; temperature = 24 ± 1°C, pH = 7, contact time = 24 h, initial As (V) concentration = 250 μg/L and adsorbent dosage = 1.2-40 g/L.

In this study, common isotherms (Langmuir and Freundlich) were employed at different doses of adsorbents (1.2–40 g/L) and at pH = 7. Equilibrium times were deducted from kinetic experiments and fixed at 24 hr. Figure 5a and 5b show the linear Freundlich and Langmuir isotherm forms for both ACP and ACZ, respectively. The correlation coefficients (R^2^) calculated for these isotherms by using linear regression procedure for adsorption of As (V) are shown in Table 4.

**Table 4:** Freundlich and Langmuir isotherm parameters

**Model**	**q**_**m**_**(mg/g)**	**b(L/mg)**	**K**_**f**_	**n**	**R**^**2**^
**Freundlich**	–	–	*0.54	*0.62	*0.98
			●0.07	●0.86	●0.99
**Langmuir**	*89.28	*5.17 × 10^-3^	–	–	*0.95
	●208.33	●3.66 × 10^-3^			●0.95

*ACP, ●ACZ.

## Discussion

Based on the XRD illustration of pumice and zeolite shown in Figure 1a and b the major constituents of pumice include hematite, quartz, ilit and mica, while the major constituents of zeolite are cilnoptilolite, quartz, mordenite and calcite. Table 1 showed that there are two major elements including SiO_2_ and Al_2_O_3_ for pumice with 51.45% and 17.08%, and for zeolite with 63.12% and 12.60%.

The surface area is an effective factor in arsenic adsorption capacity by the adsorbent .The SEM images of ACP and ACZ in Figure 1c and d showed ordered silica crystals and micro small cracks found on ACP surfaces. Also, ACZ had significant rougher surfaces than ACP. Results of the measurement of surface area can be matched with Figure 1, because the BET surface area of the ACP was measured as 7 m^2^/g, while the BET surface area of the ACZ was measured as 15 m^2^/g.

Figure 2 shows more than 95%, arsenic uptake by ACP and ACZ with initial As(V) concentration of 2000 μg/L. Also in this concentration of As(V), removal by both adsorbents have been increased rapidly from 2.5 to 20 g/L, and marginally thereafter.

When the adsorbent dose increases from 2.5 to 20 g/L, there is more specific surface for arsenate adsorption, but for higher adsorbent dosage, the removal efficiency does not increase. Also, the amount of As (V) adsorption increases when the initial concentration decreases and the adsorbent dose remains constant, because there is more specific surface for As(V) adsorption. Figure 2 also showed that ACZ was more effective than ACP for arsenic removal, because more than 96% of As (V) with initial concentration of 250 μg/L was removed by ACZ, while only 71% of As (V) could be removed by ACP with the same concentration. The reason is the larger specific surface area of ACZ in comparison with ACP. In most of the previous studies, such as arsenic adsorption onto iron oxide and aluminum [1] and natural laterite [10] as well as activated alumina impregnated with alum [15], the arsenic removal was shown to be increased by increasing the adsorbent dose and decreasing arsenic initial concentration.

The adsorption of As (V) by both adsorbents were very high at pH range of 4 to 8 and the maximum level for ACP and ACZ were at pH = 7 with the amount of 71.2% and 95.8%, respectively. Thereafter, the amount of adsorption decreased remarkably at higher pH values; only 35% and 66.1% of the arsenic adsorption by ACP and ACZ occurred at pH = 11, respectively. It is to be mentioned that the same process has been seen in the previous studies such as arsenic adsorption to iron-modified high expanded clay aggregates [4], arsenic removal by pretreated waste tea fungal biomass [21], arsenate adsorption onto iron and aluminum oxides [2], arsenic adsorption onto rare earth oxides [22] and arsenic adsorption onto activated aluminum impregnated with alum [15]. When pH increases, the adsorption decreases because the adsorption surface is negatively charged and columbic repulsions increase [10].

In the pH range of 3–11, arsenate is predominantly presented in the species of H_2_AsO_4_^-^ and HAsO_4_^2^. Therefore, it can be concluded that those are the major species being adsorbed on the surface of ACP and ACZ. The adsorption process of arsenate by both adsorbents is as equations 10–11:

(10) $$Al\;{\left(\mathrm{OH}\right)}_{3}+H^{+}+H_{2}\mathrm{As}{O_{4}}^{-}\to Al\;{\left(\mathrm{OH}\right)}_{2}-H_{2}\mathrm{As}O_{4}+H_{2}O$$

(11) $$Al\;{\left(\mathrm{OH}\right)}_{3}+2H^{+}+\mathrm{HAs}O_{4}^{2-}\to Al\;\left(\mathrm{OH}\right)-H_{2}\mathrm{As}O_{4}+2H_{2}O$$

As it is evident one of the benefits of these adsorbents is their good performance in pH value of natural waters and it is not required to use acid or alkali for adjusting pH.

In order to estimate the rate of adsorption and determine the behavior of the adsorptive, the adsorption kinetics of As(V) onto ACP and ACZ were studied at different intervals of time. Based on the results of the investigations, as shown in Table 2, during the first 5 min, 40% and 88.4% of the arsenic adsorption onto ACP and ACZ were obtained rapidly, while 58.2% and 11.4% of the adsorption for both adsorbents occurred during the next 155 min, respectively. Maximum adsorption of 95.2% and 98.8% (for ACP and ACZ, respectively) were observed at 160 min within the equilibration time.

The rate constants of As(V) adsorption were calculated by the rate expression of pseudo-first order and pseudo-second order models which have been previously described. In pseudo-first order model, the constancy rate of adsorption (K_1_) and the correlation coefficient (R^2^) for ACP and ACZ were found to be 0.0010, 0.0009, and 0.80,0.85, respectively (Figure 4a); low R^2^ shows that the adsorption of As(V) onto ACP and ACZ does not follow pseudo–first order model. In pseudo-second order model the constancy rate of adsorption (K_2_) and R^2^ for ACP and ACZ were found to be 0.0128, 0.0136, and 0.98, 0.99, respectively (Table 3). The low K_2_ and high R^2^ values suggest that the adsorption for both adsorbents to be under control of pseudo-second order model (Figure 4b). Similar rapid arsenic adsorption has been reported such as As(V) uptake by pretreated waste tea fungal biomass in which adsorption was relatively fast at the initial As(V) concentration of 4 mg/L. In this process adsorption reached the equilibrium within 90 min and the pseudo second-order model described the biosorption kinetics of As(V) with good correlation coefficient (R^2^ > 0.93) [21]. The biosorption of As(V) by *P*. *chrysogenum* reached over 70% of equilibrium uptake capacity in 10 min [23]. Arsenic removal by nanocrystalline TiO_2_ occurred rapidly and system reached equilibrium in 4 h; results were best described by pseudo-second order model (R^2^ > 0.93) [6].

In order to design an appropriate sorption system to remove As(V) from drinking water, it is important to find the well-fitted isotherm curves of ACP and ACZ. As it is evident from the R^2^ values in Table 4, the Freundlich isotherm yielded better fitting to the experimental data for both adsorbents, probably due to the heterogeneous natures of their surface sites involved in the arsenic uptake. This result also signifies that surface energy does not remain constant during the process of adsorption by ACP and ACZ, but varies with the surface coverage [24]. A similar trend has been observed in the case of phenol and 4-chlorophenol adsorptions on to pumice treated with cationic surfactant [16] and in arsenic removal from simulated industrial wastewater by magnetite nanoparticles [8]. The K_f_ and n values in Freundlich isotherm model for As(V) adsorption onto ACP and ACZ at 20°C were found to be 0.07, 0.54 and 0.86, 0.62, respectively.

The quality of water after treatment regarding the aluminum concentration was analyzed. Results showed that in maximum adsorption of As(V) at pH = 7 and 160 min by 10 g/L ACP and ACZ, 95.2% and 98.8% of arsenic was removed and reached below 12 and 3 μg/L, respectively. After treatment the soluble aluminum concentration for ACP and ACZ were 0.01 and 0.03 mg/L respectively, which is below the permissible limit set by the Institute of Standard and Industrial Research of Iran (0.2 mg/L).

Finally ACZ due to having higher porosity and specific surface area may be considered as an efficient adsorbent for providing higher adsorption capacity.

## Competing interests

The authors declare that they have no competing interests.

## Authors’ contributions

MH performed all the experiments and drafted the manuscript. SN advised the experimental methods applied and also read and edited the manuscript. Both authors read and approved the final manuscript.
